# Effect of virtual reality therapy, combined with physiotherapy for improving motor proficiency in individuals with Down syndrome: A systematic review

**DOI:** 10.4102/sajp.v77i1.1516

**Published:** 2021-05-20

**Authors:** Jessica Stander, Jennifer C. du Preez, Chantel Kritzinger, Natasha M. Obermeyer, Silke Struwig, Nikki van Wyk, Jessica Zaayman, Marlette Burger

**Affiliations:** 1 Division of Physiotherapy, Faculty of Medicine and Health Sciences, Stellenbosch University, Cape Town, South Africa

**Keywords:** virtual reality, rehabilitation, physiotherapy, occupational therapy, Down syndrome, motor proficiency

## Abstract

**Background:**

Individuals with Down syndrome may struggle with anticipatory postural adjustments, and adapt slower to motor tasks and environmental changes, due to decreased motor proficiency.

**Objectives:**

To determine the effectiveness of virtual reality therapy (VRT), specifically Nintendo Wii, combined with physiotherapy or occupational therapy (OT) for improving motor proficiency in individuals with Down syndrome, compared to standard physiotherapy, OT or no intervention.

**Method:**

Nine computerised databases were searched from inception to July 2020. Methodological quality of randomised controlled trials and quasi-experimental studies was appraised using the physiotherapy evidence database (PEDro) scale and the Joanna Briggs Institute Critical Appraisal Checklist for Case Reports.

**Results:**

Two randomised controlled trials and four quasi-experimental studies were included, with an average PEDro score of 7.3. One included case study scored 5. This review included 345 participants. Motor proficiency includes balance, coordination, strength and agility. Agility showed a significant improvement after 5 (*p* = 0.01) or 24 (*p* < 0.01) weeks. Strength showed a significant improvement after a 6- (*p* = 0.000) or 24-week intervention (*p* < 0.05). Balance showed inconclusive results for adults, and significant improvement in children after 6 (*p* = 0.000), 8 (*p* < 0.05) or 24 (*p* < 0.003) weeks. One study (*n* = 155) showed that upper limb and bilateral coordination improved significantly after 24 weeks (*p* < 0.003).

**Conclusion:**

Level II, III-1 and IV evidence suggested that VRT may be valuable to improve agility and strength in individuals with Down syndrome, and balance and coordination in children with Down syndrome.

**Clinical implications:**

It may be beneficial to use VRT in addition to standard physiotherapy or OT interventions for improving motor proficiency in individuals with Down syndrome.

## Background

Down syndrome, also known as trisomy-21, is a genetic condition that is caused by an error in cell division occurring at conception, resulting in an additional copy of chromosome 21 (Batshaw, Roizen & Pellegrino 2019). It is the most common chromosomal disorder reported in humans according to the United States of America’s National Association for Down syndrome (Presson et al. 2013). It can affect individuals of any race or ethnicity, and the overall prevalence is 10 per 10 000 live births worldwide; however, in recent years, the prevalence has been increasing (Weijerman & De Winter 2010).

Individuals with Down syndrome may present with numerous health complications (Charleton, Dennis & Marder 2010). Common anomalies include diminished muscle strength, abnormal body composition and decreased physical fitness, including reduced aerobic capacity or cardiorespiratory fitness (Baynard et al. 2008; Bertapelli et al. 2016; González-Agüero et al. 2010; Pitetti, Baynard & Agiovlasitis 2013). This may lead to low levels of resting energy expenditure and physical activity, which can result in a sedentary lifestyle (Bertapelli et al. 2016). Individuals with Down syndrome also display generalised muscle hypotonia, ligamentous laxity, articular hypermobility and difficulties in agonist–antagonist muscle co-contraction (Hardee & Fetters 2017). They struggle to perform anticipatory postural adjustments and are slower to adapt to motor task demands and environmental changes because of decreased motor proficiency (Shields, Taylor & Dodd 2008). Motor proficiency refers to the degree of skill or expertise at which gross- and fine-motor skills are executed. Total body composites included in motor proficiency include fine motor control, strength, agility, manual coordination and balance (Bruininks & Bruininks 2005).

Quality of life (QOL) decreases in individuals with Down syndrome as a result of poor motor proficiency (Zwicker, Harris & Klassen 2013). Quality of life is defined by physical, psychological and social domains (Solans et al. 2008). The physical domains include activities of daily living, such as self-care and feeding. Poor balance, coordination and agility often lead to a higher incidence of accidents, such as falls and other associated injuries (Solans et al. 2008). This, combined with their inability to coordinate fine motor movements such as holding and manipulating cutlery, may result in further disability. In the social domains, decreased QOL is observed where poor motor control often restricts these individuals to participate in community-associated activities, such as team sport and school activities. The psychological domain is also affected in terms of acceptance by peers and dependence on caregivers, leading to a lack of self-worth and accomplishment (Zwicker et al. 2013). Early commitment to physiotherapy from infancy may result in a less dependent lifestyle with greater proficiency in performing activities of daily living as they grow older (Berg et al. 2012).

Physiotherapy interventions in children with Down syndrome do not aim to accelerate gross motor development, but rather to correct or minimise compensatory strategies by improving overall motor proficiency and QOL in this population (De Morais et al. 2016). Physiotherapy interventions include approximation, strengthening, cardiovascular and balance exercises (Dodd & Shields 2005; Hardee & Fetters 2017; Li et al. 2013). Body weight support and treadmill training have also been found to accelerate walking development (De Menezes et al. 2015), whilst physiotherapy interventions based on vibration therapy have a positive effect on balance (Ruiz-González et al. 2019). The above-mentioned activities combined with family education, community integration and home-based activities aim to improve the overall function and QOL in such individuals (De Morais et al. 2016).

Alternative interventions to improve motor proficiency and overall QOL in individuals with Down syndrome include hydrotherapy, Pilates and global postural re-education (De Morais et al. 2016). A new method of rehabilitation that has caught the attention of physiotherapists is virtual reality therapy (VRT), also known as exergames (Hickman et al. 2017). Virtual reality therapy has been explored in a wide range of neurological conditions, including Parkinson’s disease, autism, cerebral palsy (CP), and patients who are affected by other developmental conditions (Hickman et al. 2017; Wang et al. 2019). A recent study concluded that VRT was effective in improving motor function in children with CP (Chen, Fanchiang & Howard 2017). Another study, investigating the impact of VRT on motor and psychosocial outcomes in children who have a developmental coordination disorder, showed a significant improvement in their motor proficiency (Hammond et al. 2014). Virtual reality therapy can also improve the spatial orientation capacity and activate the cerebral cortex, thus facilitating better balance control and motor function (Mao, Chen & Le Li 2014).

Virtual reality video games, such as Nintendo Wii Fit and Wii Sports, demand varying degrees of physical activity. Participation in VR games allows individuals to interact with displayed images, moving and manipulating virtual objects, and performing actions that immerse them in a simulated environment (Douris et al. 2012). Nintendo Wii is played with a wireless controller, fitted with acceleration sensors. This controller responds to changes in direction and speed, and interacts with the player through a motion detection system (Saposnik et al. 2010). Movements performed by the player are captured and reproduced on the screen. Feedback provided by the screen generates positive reinforcement, thus facilitating training and task improvement.

The purpose of this systematic review was to determine whether a VR therapeutic programme, specifically a Nintendo Wii-based exercise programme, alone or combined with standard physiotherapy is effective in improving motor proficiency in individuals with Down syndrome, compared with standard physiotherapy alone.

## Methodology

This systematic review was registered on PROSPERO (CRD42020190829) on 07 June 2020. This study followed reporting standards of the Preferred Reporting Items for Systematic Reviews and Meta-Analyses (PRISMA) as outlined by Moher et al. (2009). Appendix 1 provides the PRISMA checklist for systematic reviews and meta-analyses.

### Research question

The research question based on the Population, Intervention, Comparison, Outcome (PICO) format was as follows: What is the effect of VRT, alone or combined with physiotherapy or occupational therapy, compared with a standard physiotherapy or occupational therapy programme alone for improving motor proficiency in individuals with Down syndrome?

### Objectives of systematic review

The objectives of this systematic review were to:

1. determine the effect of VRT, alone or combined with physiotherapy or occupational therapy, compared with standard physiotherapy or occupational therapy alone, on motor proficiency focussing on balance, strength, coordination and agility in individuals with Down syndrome
2. critically appraise and score the identified, available randomised controlled trials (RCTs) according to the PEDro principles (https://pedro.org.au/english/resources/pedro-scale/) and available case studies according to the Joanna Briggs Institute (JBI) Critical Appraisal Checklist for Case Reports
3. describe the intervention programme for both the experimental and control groups of each included study
4. analyse and compare the results of the included studies
5. describe the outcome measures used to measure motor proficiency in individuals with Down syndrome.

### Search strategy

Nine computerised bibliographic databases, accessed through the Stellenbosch University library services, were searched, namely, MEDLINE, OTSeeker, Cochrane library, EBSCOhost, PEDro, PubMed, Science Direct, Scopus and Google Scholar. The date limit was initially set from inception up to April 2018. An update of the search was performed in June 2018 and again in June 2020. Preliminary searches within each database allowed for the elimination of unnecessary search terms, where the addition of keywords did not yield varying results. Two reviewers were assigned to each database to ensure cross-checking of results found within the different databases. All the databases were searched using the same process, and were recorded and documented. Key search terms included Down syndrome, trisomy-21, VR, motor learning, Nintendo Wii, motor proficiency, motor performance, physiotherapy, physical therapy, exercises, physical fitness and functional mobility. The detailed search strategies, specifically developed for each database according to its functions, are provided in Appendix 2.

### Study selection

**FIGURE 1:**
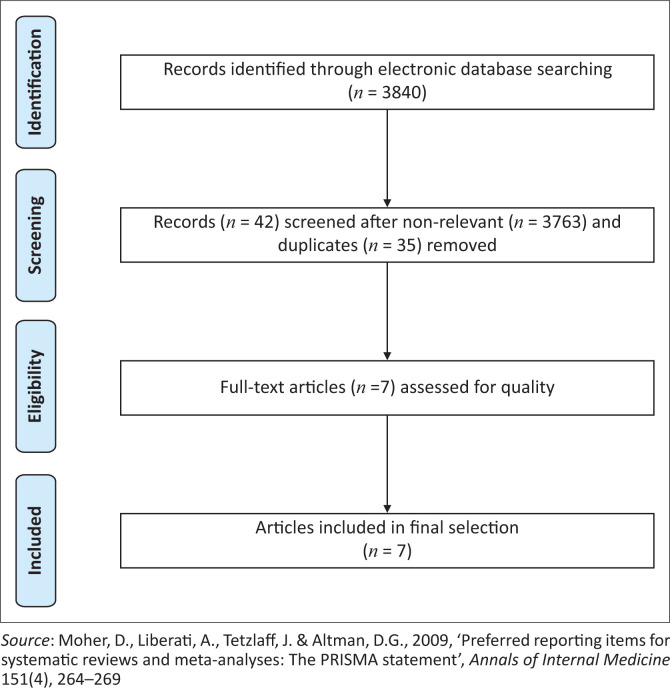
Preferred Reporting Items for Systematic Reviews and Meta-Analyses flow diagram.

Each of the reviewers independently searched two randomly selected databases and screened titles according to the eligibility criteria of the review. Thereafter reviewers compared results and eliminated duplicate titles. Abstracts for all selected titles were retrieved, and each reviewer independently screened the abstracts against the eligibility criteria. In the case where consensus could not be reached, the abstracts of those titles that could not be agreed upon were retrieved and assessed using the eligibility criteria. In the case of persisting disagreement, the rest of the reviewers or J.S. or M.B. were consulted to reach consensus. Full-text articles, from the selected abstracts, were subsequently retrieved and were independently screened for eligibility by each reviewer. The reviewers compared the eligible full texts identified for inclusion, and if consensus regarding the final inclusion of articles was not reached, J.S. or M.B. was contacted to resolve the matter. The search method is illustrated in Figure 1.

### Criteria for considering studies

#### Types of studies

Randomised controlled- or clinical-trials, non-randomised controlled- or clinical-trials, pilot and case studies published in English from inception of the database until July 2020 were considered for inclusion in this systematic review. Including studies with control groups with no active intervention was a change to the original protocol and deemed to be appropriate if the control group had pre–post assessment of the same outcome measures.

#### Types of participants

Study participants with Down syndrome included were children and adults (6–60 years). Participants were excluded from the review if they had additional neuro-musculoskeletal disorders and/or severe sensory (visual and auditory) impairments.

#### Types of interventions

Studies in which participants received VRT, alone or combined with physiotherapy or OT.

#### Types of comparison

Control groups had to receive standard physiotherapy care, occupational therapy care or no intervention.

#### Types of outcome measures

Any valid outcome measures of motor proficiency (i.e. balance, strength, coordination and agility) were included in the review:

- balance, for example, Bruininks-Oseretsky Test of Motor Proficiency, Second edition (BOT-2); Flamingo balance test; Paediatric balance test; Timed up-and-go test; Five-times-sit-to-stand test; and Pressure sensing mat systems
- agility, for example, shuttle run test; BOT-2; Test of Gross Motor Development, Second Edition (TGMD-2)
- strength, for example, hand-grip test, 30-s sit-up; standing broad jump test
- coordination, for example, Bruininks-Oseretsky Test of Motor Proficiency, Second edition; TGMD-2.

#### Evidence hierarchy

The National Health and Medical Research Council (NHMRC) Evidence Hierarchy was used to appraise each of the articles identified and considered for the study, and was, thus, needed to ensure validity and reliability of the included articles (Merlin, Weston & Tooher 2009). The reviewers discussed and justified the NHMRC score for each article, where consensus was not reached amongst group members; J.S. or M.B. were consulted.

#### Methodological appraisal

Reviewers individually scored each article, where after discrepancies were discussed within the review group and resolved by contacting J.S. or M.B. The methodological quality of each of the included RCTs was determined by critical appraisal using the Physiotherapy Evidence Database (PEDro) scale, aimed at establishing the methodological quality of an RCT (De Morton 2009; Verhagen et al. 1998). The reliability of the PEDro scale was tested and reported to be acceptable (Maher et al. 2003). The PEDro scale evaluates RCTs based on 11 specific criteria. Each criterion is given a score of 1 (present) or 0 (absent), with the total score being a maximum of 11 (De Morton 2009; Maher et al. 2003). The higher the RCT scores out of a total of 11, the higher the methodological quality of the RCT. The reviewers were divided into pairs to appraise the RCTs and compare their scores using the PEDro scale. Methodological quality of the case study was appraised using the JBI Critical Appraisal Checklist for Case Reports (Gagnier et al. 2013). The checklist has eight questions with yes, no, unclear or not applicable to determine whether the case report is of good quality (Appendix 3). Where there was discrepancy in the final scores, the authors consulted the group members. Where consensus was still not reached, J.S. or M.B. was consulted to assist with the final score.

#### Data extraction

Data were extracted from each of the selected articles using the adapted JBI Data Extraction Form for Systematic Review of Experimental studies (Pearson, Field & Jordan 2009) (available from the corresponding author upon request). The data were extracted under the following headings: citation, type of study, participants (including number of participants and ages), interventions, comparisons, outcome measures (including measurement tool, validity and reliability), dichotomous data (intervention and comparison group), continuous data (intervention and comparison group), clinical status post-intervention and implication thereof. The articles were divided amongst the reviewer team. Each article was assigned to two reviewers, who were responsible for independently extracting the necessary data. Where reviewers failed to find all the required information within the data extraction form, the corresponding authors of the articles were contacted via email to obtain the missing data. After the data extraction, data findings were compared among group members, ultimately reaching consensus whether the obtained data were correct and complete. Where review members failed to reach consensus, J.S. or M.B. was consulted.

#### Data analysis

Following the data extraction process, the critical analysis of the data was performed by the reviewers. Each article was reviewed by at least two reviewers. The two reviewers responsible for an article independently analysed the data, thereafter, compared their findings. Contrasted findings were shared and discussed with the rest of the group. J.S. or M.B. were consulted for the final decision where consensus was not reached amongst group members. Statistical pooling was not possible because of the heterogeneous nature of the data and the intervention, and follow-up periods. The results are presented in a narrative form and illustrated in tables.

## Results

### Search results and description of studies

A total of 3840 initial hits were found. Of these, 42 titles and abstracts were considered, and 35 of these abstracts were duplicates, leaving seven eligible full-text articles for quality assessment, for use in this systematic review. Figure 1 graphically depicts the PRISMA flow diagram of article selection and inclusion (Moher et al. 2009).

### Evidence hierarchy

The final seven eligible articles that were used in this systematic review are two RCTs (Ghafar & Abdelraouf 2017; Lin & Wuang 2012), four quasi-experimental studies (Álvareza et al. 2018; Rahman 2010; Silva et al. 2017; Wuang et al. 2011) and a case study (Berg et al. 2012). According to the NHMRC Hierarchy of evidence (Merlin et al. 2009), the articles are classified as level II evidence (Ghafar & Abdelraouf 2017; Lin & Wuang 2012), level III-1 evidence (Álvareza et al. 2018; Rahman 2010; Silva et al. 2017; Wuang et al. 2011) and level IV evidence (Berg et al. 2012).

### Methodological appraisal

The methodological quality of six experimental studies was assessed using the 11-item PEDro scale (Maher et al. 2003) and scored between six and nine, with an average score of 7.3 out of 11. Table 1 provides a brief summary of the individual article’s scores on the PEDro scale. During the methodological appraisal of the final articles, it was found that criteria 5 (blinding of the participants) and criteria 6 (blinding of the therapists) were not fulfilled in any of the six studies. The case study by Berg et al. (2012) scored 6/8 on the JBI Critical Appraisal Checklist for Case Reports (Gagnier et al. 2013). The two criteria that Berg et al. (2012) failed to fulfil included criteria 1 (clearly described demographic characteristics) and criteria 2 (clearly described history).

**TABLE 1:** Methodological quality of included studies.

S. no.	PEDro criteria	Álvareza et al. (2018)	Ghafar and Raouf (2017)	Lin and Wuang (2012)	Rahman (2010)	Silva et al. (2017)	Wuang et al. (2011)
1	Eligibility criteria were specified.	Yes	Yes	Yes	Yes	Yes	Yes
2	Subjects were randomly allocated to groups (in a crossover study, subjects were randomly allocated an order in which treatments were received).	Yes	Yes	Yes	No	Yes	Yes
3	Allocation was concealed.	No	No	No	No	No	No
4	The groups were similar at baseline regarding the most important prognostic indicators.	Yes	Yes	Yes	Yes	Yes	Yes
5	There was blinding of all subjects.	No	No	No	No	No	No
6	There was blinding of all therapists who administered the therapy.	No	No	No	No	No	Yes
7	There was blinding of all the assessors who measured at least one key outcome.	No	Yes	Yes	No	Yes	Yes
8	Measures of at least one key outcome were obtained for more than 85% of the subjects initially added to the groups.	Yes	Yes	Yes	Yes	Yes	No
9	All subjects for whom outcome measures were available received the treatment or control condition as allocated or, where this was not the case, data for at least one key outcome was analysed by ‘intention to treat’.	Yes	Yes	Yes	Yes	Yes	No
10	The results of between-groups statistical comparisons are reported for at least one key outcome.	Yes	Yes	Yes	Yes	Yes	Yes
11	The study provides both point measures and measures of variability for at least one key outcome.	Yes	Yes	Yes	Yes	Yes	Yes
	**Total/11**	**7**	**8**	**8**	**6**	**8**	**7**

*Source:* Maher, C.G., Sherrington, C., Herbert, R.D., Moseley, A.M. & Elkins, M., 2003, ‘Reliability of the PEDro Scale for rating quality of randomized controlled trials’, *Physical Therapy* 83(8), 713–721

### Study sample description

Sample descriptions for each study are summarised in Table 2. The seven studies had a wide variation in sample size, contributing to a total sample size of 345 participants, with 148 in the experimental group. The studies included participants between the ages of 6 and 60 years. Wuang et al. (2011), Silva et al. (2017) and Ghafar and Abdelraouf (2017) did not specify the number of male and female participants, Rahman (2010) and Lin and Wuang (2012) had more female participants, and Álvareza et al. (2018) had more male participants. The study by Berg et al. (2012) had one male participant. No baseline differences of participants were reported in any of the studies. The studies were conducted in Saudi Arabia (Ghafar & Abdelraouf 2017; Rahman 2010), Taiwan (Lin & Wuang 2012; Wuang et al. 2011), Chile (Álvareza et al. 2018) and Portugal (Silva et al. 2017).

**TABLE 2:** Study sample description.

Criteria	Groups	Álvareza et al. (2018)	Ghafar and Abdelraouf (2017)	Lin and Wuang (2012)	Rahman (2010)	Silva et al. (2017)	Wuang et al. (2011)	Berg et al. (2012)
Sample size (*n*)	Experimental	*n* = 9	*n* = 13	*n* = 46	*n* = 15	*n* = 12	*n* = 52	*n* = 1
	Control	*n* = 7	*n* = 13	*n* = 46	*n* = 15	*n* = 13	*n* = 103 (SOT = 53; No intervention = 50)	-
Gender (*n*)	Experimental	Female = 3 Male = 13	Not specified	Female = 25 Male = 21	Female = 9 Male = 6	Not specified	Not specified	Male
	Control	-	Not specified	Female = 24 Male = 22	Female = 8 Male = 7	Not specified	Not specified	-
Age mean (SD) (years)	Experimental	8.30 ± 2.06	7.18 ± 1.85	15.6 ± 3.6	10.92 ± 1.16	18–60 years	7–12 years	12-year-old
	Control	8.43 ± 1.62	7.40 ± 1.27	14.9 ± 3.9	11.56 ± 0.44	18–60 years	7–12 years	-
Baseline difference reported	Experimental	No baseline differences reported	No baseline differences reported	No baseline differences reported	No baseline differences reported	No baseline differences reported	No baseline differences reported	Not applicable
	Control	No baseline differences reported	No baseline differences reported	No baseline differences reported	No baseline differences reported	No baseline differences reported	No baseline differences reported	Not applicable
Country of study	-	Chile	Saudi Arabia	Taiwan	Saudi Arabia	Portugal	Taiwan	USA
Type of study	-	Quasi-experimental	Randomised controlled trial	Randomised controlled trial	Quasi-experimental	-	Quasi-experimental	Case study

SD, standard deviation; SOT, standard occupational therapy; USA, United States of America.

### Description of intervention and control

Table 3 summarises the interventions used across the seven studies’ experimental and control groups. Variations in the type and/or dosages of the Nintendo Wii games used were evident across the studies. Three studies used both Nintendo Wii Fit and Sports (Berg et al. 2012; Ghafar & Abdelraouf 2017; Wuang et al. 2011), and four studies (Álvareza et al. 2018; Lin & Wuang 2012; Rahman 2010; Silva et al. 2017) used Nintendo Wii fit; however, the same games and activities were included in the different Nintendo Wii programmes. Standard physiotherapy or occupational therapy interventions were similar across the included studies, as described below. The control groups of Lin and Wuang (2012) and Álvareza et al. (2018) had no intervention reported, whilst Berg et al. (2012) did not include a control group.

**TABLE 3:** Description of intervention and control procedures.

Groups	Variables	Álvareza et al. (2018)	Ghafar and Abdelraouf (2017)	Lin and Wuang (2012)	Rahman (2010)	Silva et al. (2017)	Wuang et al. (2011)	Berg et al. (2012)
Experimental group: Nintendo Wii Games	Type of Nintendo Wii Games	Wii fit software, along with the Wii balance board. The following games were practised: Snowboarding, the Penguin Slide, Super Hoola Hoop, heading Soccer and Skii Jumping.	Wii Sports, Wii Fit, Wii balance board. The following three Wii games were practised: Football heading game, Ski Slalom game and, finally, the Table Tilt game.	Wii Sports games. The five most popular games being Boxing, bowling, table tennis, Frisbee and golfing.	Wii Fit with Wii balance board, Wii console, Wii remote and Wii nun chuck. Balance games: Soccer heading game, Tightrope walk game, Penguin slide game As well as the following: Approximation and strengthening exercises, walking on an even surface in the treatment room and climbing stairs.	Wii fit balance board. Games targeting balance or isometric strength: Freerun, Heading, Snowboard Slalom, Table tilt, Tight rope tension, Hoolahoop, Balance bubble, Penguin slide Wii Sports, Wii Sports Resort, Wii Fit and Just Dance 2 targeted aerobic endurance. Games performed: Sword play, Boxing, Cycling, Table tennis, Just Dance 2.	A Nintendo Wii gaming console, that is, Wii Sports. No games specified.	Wii Sports bowling, baseball, rhythm boxing and snowboarding game.
	Frequency and dosage of intervention	The intervention was 5 weeks with two weekly sessions of 20-min duration.	Treatment sessions consisted of 30 min, three times per week for 8 weeks. Each game was played for 10 min.	Treatment sessions consisted of three 35-min sessions per week for 6 weeks.	Approximation and strengthening exercises (15 min, 5-min rest) Walking on even surfaces and climbing stairs for 35 min (15 min, 5-min rest). Wii game practised for 5 min, with 5 min rest in-between. In the order: football heading game, tight rope game and penguin slide game. Programme performed twice a week for 6 weeks.	Participants completed 1 h session, three times per week, to 22 sessions over a period of 2 months.	Treatment sessions consisted of 1 h, 2 days per week for 24 weeks.	Participant asked to use Wii 20 min, four times per week for 8 weeks.
Control group: SPT; SOT; No intervention	Method of SPT or SOT	Continued normal daily activities along with psycho-educational therapies included in school.	Throwing or catching balls or beanbags outside of their base of support, reaching for objects whilst standing or sitting on stable or unstable surfaces, walking up and down stairs, balance beam walk, single-leg stance and kicking activities. Individualised treatments based on functional limitations and abilities of child.	No intervention.	Approximation or strengthening exercises. Walking on an even surface in the treatment room and climbing stairs.	The control group completed their usual daily activities, such as vocational rehabilitation, life-skill training and art-related activities.	Various activity combinations incorporating the principle of sensory integrative therapy, neuro developmental treatment, and perceptual motor approaches.	Not applicable.
	Frequency and dosage of intervention	None reported.	Treatment sessions consisted of 30 min, three times per week for 8 weeks.	None reported.	Approximation or strengthening exercises (15 min, 5 min rest). Walking on even surfaces and climbing stairs for 35 min (15 min each and 5 min rest in between). Programme performed twice a week for 6/52.	None was mentioned or specified.	Treatment sessions consisted of 30 min, three times per week for 8 weeks.	Not applicable.

SPT, standard physiotherapy; SOT, standard occupational therapy.

### Description of outcome measures

The outcome measures used within the included studies assessed balance, agility, strength and coordination. The formal explanation for each outcome measure is described in Appendix 4.

Balance was the only outcome that was measured across most of the included studies. Rahman (2010), Berg et al. (2012) and Wuang et al. (2011) used the BOT and the BOT-2. Ghafar and Abdelraouf used the Paediatric Balance Scale, which is a modified version of the Berg Balance Scale (PBS). This study also made use of the timed-up-and-go test, as well as the five-times-sit-to-stand test, to measure balance. Both these tests have been found to be reliable and valid with the measurement of balance in children with disabilities, as well as measuring balance in typically developing children (Beerse, Lelko & Wu 2019; Kumban et al. 2013). The data collected from the outcome measures were recorded at baseline, as well as at the end of the training programme at 8 weeks. Álvareza et al. (2018) used the TGMD-2 and area of movement of the pressure centre with eyes open and closed, as determined by the Wii balance board.

Silva et al. (2017) used the beanbag overhead throw to test coordination and used the hand grip test and standing broad jump test to measure strength. Wuang et al. (2011), Berg et al. (2012) and Lin and Wuang (2012) used the BOT-2 agility and strength subsections to test agility and strength, respectively. However, Wuang et al. (2011) tested at baseline and 24-week follow-up, whereas Lin and Wuang (2012) tested at baseline and 6-week follow-up and Berg et al. (2012) tested at baseline and 8-week follow-up. Both Wuang et al. (2011) and Berg et al. (2012) used the BOT-2 coordination subset at baseline and at 24- and 8-week follow-up, respectively.

### The effect of virtual reality therapy combined with physiotherapy

The effect of VRT, alone or combined with physiotherapy, on motor proficiency (including balance, agility, coordination and strength) in children and adults with Down syndrome compared with standard physiotherapy is discussed under the following subheadings:

### Balance

Table 4 summarises the mean scores from baseline to follow-up for the included studies. Álvareza et al. (2018) reported no significant improvement in balance when assessing pressure centre changes with both eyes open and closed (*p* = 0.31 and *p* = 0.13, respectively). However, there was a significant within-group improvement in balance when assessing pressure centre changes with eyes closed (*p* = 0.039). Ghafar and Abdelraouf (2017) reported a significant improvement in balance for both the paediatric balance and timed-up-and-go tests (*p* = 0.046 and *p* = 0.043, respectively). Both Rahman (2010) and Wuang et al. (2011) used the BOT-2 balance subsection outcome measure. Rahman (2010) reported a significant increase in balance (within group analysis) at the 6-week follow-up, whilst the between-group analysis demonstrated a significant change in favour of the experimental group (*p* = 0.000). Wuang et al. (2011) reported a higher mean change and greater effect size of the experimental group when compared with the control group at follow-up after 24 weeks of intervention. There was, however, no significant difference (*p* > 0.05) between groups at follow-up. Silva et al. (2017) assessed balance using the Flamingo Balance Test. Neither the control nor the experimental group displayed a significant within-group effect size. The *p*-value (*p* = 0.477) also shows no significant change between the two groups at the 24-week follow-up. Berg et al. (2012) reported a small improvement in BOT balance subtest scores (mean change = 1; minimum detectable change = 1.14; minimum important difference = 0.57).

**TABLE 4:** Results reported for balance measures of included studies.

Study	Assessment interval	Test description	Control		Experimental group		Mean difference between groups (95% CI)	*p*
			mean	(SD)	mean	(SD)		
Álvareza et al. (2018)	Baseline	Pressure centre eyes open (m^2^)	0.06	0.05	0.06	0.04	0.00 (−0.05 to 0.05)	0.83
	5 weeks		0.04	0.03	0.07	0.005	0.03 (0.01 to 0.05)	0.31
	*p*-value (within group)		0.36	-	0.52	-	-	-
	Sample size (*n*)		7	-	9	-	-	-
	Baseline	Pressure centre eyes closed (m^2^)	0.05	0.02	0.05	0.03	0.00 (−0.03 to 0.03)	0.86
	5 weeks		0.04	0.02	0.02	0.019	−0.02 (−0.04 to 0.00)	0.13
	*p*-value (within group)		0.31	-	0.039*	-	-	-
	Sample size (*n*)		7	-	9	-	-	-
Ghafar and Abdelraouf (2017)	Baseline	Pediatric balance test	47.35	3.8	48.2	4.6	0.85 (−2.57 to 4.27)	0.046
	8 weeks		52.15	4.7	57.75	2.6	5.60 (2.53 to 8.67)	-
	*p*-value (within group)		Not reported	-	Not reported	-	-	-
	Sample size (*n*)		13	-	13	-	-	-
	Baseline	Timed up and go test	10.65	1.7	10.21	2.0	−0.44 (−1.94 to 1.06)	0.043
	8 weeks		8.95	1.4	7.01	1.8	1.94 (0.63 to 3.25)	-
	*p*-value (within group)		Not reported	-	Not reported	-	-	-
	Sample size (*n*)		13	-	13	-	-	-
	Baseline	Five times sit to stand test	16.56	2.3	15.6	2.6	−0.96 (−2.95 to 1.03)	0.027
	8 weeks		14.62	3.2	11.2	2.9	−3.42 (−5.89 to −0.95)	-
	*p*-value (within group)		Not reported	-	Not reported	-	-	-
	Sample size (*n*)		13	-	13	-	-	-
Rahman (2010)	Baseline	BOT-2 balance subsection	8.87	5.53	10.27	4.83	1.4 (−2.48 to 5.28)	0.466
	6 weeks		10.40	4.93	17.47	3.50	7.01 (3.87 to 10.27)	0.000
	*p*-value (within group)		0.017	-	0.000	-	-	-
	Sample size (*n*)		15	-	15	-	-	-
Silva et al. (2017)	Baseline	Flamingo balance test	3.31	8.20	6.08	11.09	2.77 (−5.25 to 10.79)	0.477
	24 weeks		1.69	6.10	9.92	12.53	8.23 (0.18 to 16.28)	-
	Effect size – within group (*d*)		0.228	-	0.372	-	-	-
	Sample size (*n*)		13	-	12	-	-	-
Wuang et al. (2011)	Baseline	BOT-2 balance subsection	11.40	8.91	11.08	7.02	−0.32 (−3.43 to 2.79)	< 0.003
	24 weeks		12.66	7.99	13.27	8.91	0.61 (−2.66 to 3.88)	-
	Effect size – within group (*d*)		0.72	-	1.60	-	-	-
	Sample size (*n*)		53	-	52	-	-	-

SD, standard deviation; CI, confidence interval; BOT-2, Bruininks–Oseretsky Test of Motor Proficiency, Second edition.

### Agility

Table 5 summarises the mean scores from baseline to follow-up for the included studies. Álvareza et al. (2018) reported no significant improvement in agility when assessing locomotion as part of the TGMD-2 for between-group or within-group results. Both Wuang et al. (2011) and Lin and Wuang (2012) used the BOT-2 running speed and agility subsection. Lin and Wuang (2012) reported a significant change (*p* = 0.01) favouring the experimental group after the 6-week intervention. Wuang et al. (2011) reported that the experimental group had a greater effect size within the group when compared with the control group. The *p*-value (*p* < 0.003) confirms that the change between groups is significant and favours the experimental group when referring to the BOT-2 running speed and agility subsection. Silva et al. (2017) made use of the shuttle run test, one item of the BOT-2 running speed and agility subsection, for assessing the change in mean scores at follow-up after a 24-week intervention. As displayed in Table 5, running speed and agility decreased in the control group, whilst it increased in the experimental group at 24 week intervention. Between-group analysis showed a significant change (*p* = 0.014) in favour of the experimental group. Berg et al. (2012) reported a small improvement in BOT agility subtest scores (mean change = 1; minimum detectable change = 1.14; minimum important difference = 0.59).

**TABLE 5:** Results reported for agility measures of included studies.

Study	Assessment interval	Test description	Control		Experimental group		Mean difference between groups (95% CI)	*p*
			mean	(SD)	Mean	(SD)		
Álvareza et al. (2018)	Baseline	TGMD-2 locomotion subsection	33.71	3.69	34.56	5.94	0.85 (−5.85 to 7.55)	0.75
	5 weeks		33.71	4.82	36.67	3.39	2.96 (−1.43 to 7.35)	0.17
	*p*-value (within group)		1.00	-	0.3	-	-	-
	Sample size (*n*)		7	-	9	-	-	-
Lin and Wuang (2012)	Baseline	BOT-2 agility subsection	11.0	5.9	11.0	6.3	0.00 (−2.53 to 2.53)	0.466
	6 weeks		10.0	6.8	16.0	6.6	6.0 (3.22 to 8.78)	0.01
	*p*-value (within group)		Not reported	-	Not reported	-	-	-
	Sample size (*n*)		46	-	46	-	-	-
Silva et al. (2017)	Baseline	Shuttle run test	33.01	5.69	35.42	12.55	2.41 (−5.54 to 10.36)	0.014
	24 weeks		35.31	9.06	31.62	6.32	−3.69 (−10.21 to 2.83)	-
	Effect size – within group (*d*)		0.508	-	0.478	-	-	-
	Sample size (*n*)		13	-	12	-	-	-
Wuang et al. (2011)	Baseline	BOT-2 agility subsection	7.47	5.58	7.38	5.48	−0.09 (−2.23 to 2.05)	< 0.003
	24 weeks		9.36	6.81	10.12	5.64	0.76 (−1.66 to 3.18)	-
	Effect size – within group (*d*)		1.89	-	2.56	-	-	-
	Sample size (*n*)		53	-	52	-	-	-

SD, standard deviation; CI, confidence interval; BOT–2, Bruininks–Oseretsky Test of Motor Proficiency, Second edition; Test of Gross Motor Development, Second Edition.

### Strength

Table 6 summarises the mean scores from baseline to follow-up for the included studies. Both Wuang et al. (2011) and Lin and Wuang (2012) used the BOT-2 strength subsection as the outcome measure. Lin and Wuang (2012) reported a significant change (*p* = 0.02) favouring the experimental group after the 6-week intervention. The change in mean scores for the BOT-2 strength subsection from baseline to follow-up at 24-week intervention of Wuang et al. (2011) is tabulated in Table 6. Although the effect size within group post 24-week intervention is greater for the experimental group compared with the control group, the between-group analysis was not significant (*p* > 0.05). Table 6 also summarises the following results for Silva et al. (2017): change in the mean score, standard deviation (SD), the within-group size effect and the *p*-value for the handgrip test, 30 s sit-up and standing broad jump test at baseline and follow-up at 24-week intervention. The handgrip test showed no significant difference between the control and experimental group at 24-week intervention (*p* = 0.837). At 24 weeks, significant change was observed between the groups for the 30-s sit-up (*p* = 0.04) and the standing broad jump test (*p* < 0.003) favouring the experimental group. Berg et al. (2012) reported no improvement in BOT strength subtest scores (mean change = 0; minimum detectable change = 1.47; minimum important difference = 1.73).

**TABLE 6:** Results reported for strength measures of included studies.

Study	Assessment interval	Test description	Control		Experimental group		Mean difference between groups (95% CI)	*p*
			mean	(SD)	mean	(SD)		
Lin and Wuang (2012)	Baseline	BOT-2 strength subsection	10.94	1.59	10.69	1.25	−0.25 (−0.84 to 0.34)	0.466
	6 weeks		14.36	1.87	15.37	1.80	1.01 (0.25 to 1.77)	0.000
	*p*-value (within group)		Not reported	-	Not reported	-	-	-
	Sample size (*n*)		46	-	46	-	-	-
Silva et al. (2017)	Baseline	Handgrip test	22.38	5.91	23.67	6.89	1.29 (−4.01 to 6.59)	0.837
	24 weeks		23.92	6.45	25.42	5.53	1.5 (−3.49 to 6.49)	-
	Effect size – within group (*d*)		0.693	-	0.618	-	-	-
	Sample size (*n*)		13	-	12	-	-	-
	Baseline	30-s sit-up	9.96	5.44	7.17	5.51	−2.79 (−7.32 to 1.74)	0.040
	24 weeks		7.69	5.22	8.00	5.36	0.21 (−4.17 to 4.59)	-
	Effect size – within group (*d*)		0.585	-	0.271	-	-	-
	Sample size (*n*)		13		12	-	-	-
	Baseline	Standing broad jump	88.04	44.02	82.67	31.52	−5.37 (−37.29 to 26.55)	0.003
	24 weeks		90.69	35.20	99.33	29.49	8.64 (−18.35 to 35.63)	-
	Effect size – within group (*d*)		0.235	-	1.691	-	-	-
	Sample size (*n*)		13	-	12	-	-	-
Wuang et al. (2011)	Baseline	BOT-2 strength subsection	10.94	8.14	10.69	6.40	−0.25 (3.09 to 2.59)	< 0.003
	24 weeks		14.36	9.58	15.37	9.22	1.01 (−2.63 to 4.65)	-
	Effect size – within group (*d*)		2.15	-	3.74	-	-	-
	Sample size (*n*)		53	-	52	-	-	-

SD, standard deviation; CI, confidence interval; BOT-2, Bruininks–Oseretsky Test of Motor Proficiency, Second edition.

### Coordination

Table 7 summarises the mean scores from baseline to follow-up for the included studies. Álvareza et al. (2018) reported no significant improvement in balance when assessing manipulation as part of the TGMD-2 between the control and experimental groups (*p* = 0.07). However, there was a significant within-group improvement in coordination (*p* = 0.01). Silva et al. (2017) assessed coordination using the Beanbag Overhead throw test. Also in Table 7, the mean scores and SD for the Beanbag overhead throw test, both left and right hand, are tabulated from baseline to follow-up at 24-week intervention. When looking at the right-handed overhead throw, no significant change (*p* > 0.05) between the groups was observed. However, for the left-handed overhead throw, significant change (*p* = 0.010) was observed within the experimental group from baseline to 24-week. Furthermore, the *p*-value (*p* > 0.05) for the between-group difference does not signify any significant change at the 24-week intervention. The change in mean scores of Wuang et al. (2011) for the BOT-2 subsection testing coordination from baseline to follow-up at 24-week intervention is tabulated in Table 7. In the upper limb and bilateral coordination subsection for the BOT-2, the between-group change at a 24-week follow-up is significant (*p* < 0.003) favouring the experimental group. Berg et al. (2012) reported only an improvement in BOT upper-limb coordination subtest scores (mean change = 1; minimal detectable change [MDC] = 1.70; minimal important difference [MID] = 1.61). Berg et al. (2012) also reported no improvement in BOT bilateral coordination subtest scores (mean change = −6; minimum detectable change = 1.52; minimum important difference = 1.11).

**TABLE 7:** Results reported for coordination measures of included studies.

Study	Assessment interval	Test description	Control		Experimental group		Mean difference between groups (95% CI)	*p*
			mean	(SD)	mean	(SD)		
Álvareza et al. (2018)	Baseline	TGMD-2 manipulation subsection	30.14	6.67	28.44	5.46	−1.7 (−8.19 to 4.79)	0.58
	5 weeks		29.43	5.86	35.00	5.50	5.57 (−0.54 to 11.68)	0.07
	*p*-value (within group)		0.09	-	0.01*	-	-	-
	Sample size (*n*)		7	-	9	-	-	-
Silva et al. (2017)	Baseline	Bean bag overhead throw test: Right hand	6.69	3.38	5.17	3.76	−1.52 (−4.47 to 1.43)	0.150
	24 weeks		5.23	2.89	6.67	3.11	1.44 (−1.042 to 3.92)	-
	Effect size – within group (*d*)		0.478	-	0.591	-	-	-
	Sample size (*n*)		13	-	12	-	-	-
	Baseline	Beanbag overhead throw test: Left hand	8.15	3.76	6.92	3.53	−1.23 (−4.25 to 1.79)	0.083
	24 weeks		5.38	3.15	6.67	3.37	1.29 (−1.4 to 3.99)	-
	Effect size – within group (*d*)		0.635	-	0.010	-	-	-
	Sample size (*n*)		13	-	12	-	-	-
Wuang et al. (2011)	Baseline	Upper limb coordination	8.11	1.12	7.96	1.14	−0.15 (−0.59 to 0.29)	<0.003
	24 weeks		9.32	2.44	10.62	2.64	1.3 (0.32 to 2.28)	-
	Effect size – within group (*d*)		1.08	-	2.33	-	-	-
	Sample size (*n*)		53	-	52	-	-	-
	Baseline	Bilateral coordination	10.94	8.14	10.69	6.40	−0.25 (−3.09 to 2.59)	< 0.003
	24 weeks		14.36	9.58	15.37	9.22	1.01 (−2.63 to 4.65)	-
	Effect size – within group (*d*)		0.96	-	1.90	-	-	-
	Sample size (*n*)		53	-	52	-	-	-

SD, standard deviation; CI, confidence interval; BOT-2, Bruininks–Oseretsky test of Motor Proficiency, Second edition.

### Ethical considerations

This article followed all ethical standards for research without direct contact with human or animal subjects.

## Discussion

To the authors’ knowledge, our study is the first systematic review conducted in English on the effectiveness of VRT, specifically using Nintendo Wii, alone or combined with physiotherapy or occupational therapy, compared with standard physiotherapy or occupational therapy alone or no intervention, on motor proficiency in individuals with Down syndrome. The only literature review that was found was conducted by De Menezes et al. (2015) in Portuguese, which did not meet the inclusion criteria of our systematic review. The abstract from De Menezes et al. (2015) suggested that VRT may be of benefit along with physiotherapy to improve motor proficiency (De Menezes et al. 2015).

The main outcome assessed was motor proficiency, which consists of the following components: balance, strength, coordination and agility. Of the included studies, only Rahman (2010) and Ghafar and Abdelraouf (2017) showed significant improvements in balance within the experimental group, compared with the control group. The PBS, employed by Ghafar and Abdelraouf (2017), is specifically used to assess balance in young children who present with mild-to-moderate disabilities, and it has already been established that the PBS is a reliable and valid tool for the measurement of balance (Franjoine, Gunther & Taylor 2003). Álvareza et al. (2018) reported a significant within-group improvement in pressure centre (eyes closed) change in the experimental group. Both Wuang et al. (2011) and Silva et al. (2017) documented no significant improvements in balance when comparing the experimental group with the control group using the Flamingo balance test and the BOT-2 subtest, respectively. Bruininks–Oseretsky Test of Motor Proficiency, Second Edition is a valid outcome measure for this population group, as it has a satisfactory agreement with other motor performance measures, namely, the Peabody Developmental Motor Scale, Second Edition (Folio & Fewell 2000) and the Test of Visual Motor Skills-Revised (Gardner 1995). The BOT-2 is suitable to assess motor proficiency in children with intellectual disability, with an excellent reliability ((intra-class correlation coefficient [ICC] = 0.99) (Wuang & Su 2009). There is evidence which suggest that balance, amongst other things, weakens with the ageing process (Iwasaki & Yamasoba 2015). This could be a possible reason why the results for balance differed greatly among these studies. Silva et al. (2017) reported a decline in scores from baseline to 24-week intervention follow-up. The only differences found among the included studies were the variation in outcome measures and the duration of interventions. The case study conducted by Berg et al. (2012), using an 8-week VRT-based intervention for individuals with Down syndrome, reported similar findings compared with Rahman (2010) and Ghafar and Abdelraouf (2017) on improvement in balance. These findings are important as all of these included studies reported small-to-medium improvement changes in balance. There has been a considerable increase in the life expectancy of individuals with Down syndrome over the last few decades (Weijerman & De Winter 2010). As individuals with Down syndrome, now, have an improved life expectancy, it is important to focus on treatment regimens that will contribute to the improvement of balance. Balance is a key component in the activities of daily living and adds to their QOL, as it allows them to interact in social activities, including sports, with their peers. Without these balance reactions, the individual with Down syndrome will resolve to a more sedentary lifestyle, which facilitates other complications, such as decreased cardiovascular fitness and reduced aerobic capacity (Bertapelli et al. 2016).

Silva et al. (2017) and Wuang et al. (2011) found a significant improvement in the agility outcome within the experimental group, compared with the control group, at a 24-week intervention. Silva et al. (2017) used the shuttle run test as an outcome measure, and Wuang et al. (2011) used the speed and agility subtest of the BOT-2. Both studies used the same duration of intervention, namely, 1-h sessions. Silva et al. (2017) performed these sessions three times per week, whilst Wuang et al. (2011) performed twice. Álvareza et al. (2018) found non-significant changes in agility outcomes, but this study utilised the TGMD-2 locomotion subsection which is different from the other included studies. The TGMD-2 has been shown to have high reliability (Ulrich & Sanford 2000).

Other studies carried out by Lin and Wuang (2012) and Berg et al. (2012) documented significant findings after a shorter intervention period. Lin and Wuang (2012), who combined Wii Sport games with treadmill exercises to engage adolescents with Down syndrome in a 6-week agility and strength training programme reported a significant improvement in the experimental group. Berg et al. (2012), a case report on the motor control outcomes of Nintendo Wii, also reported improvement in agility after an 8-week intervention. Agility improvements will lead to quicker responses and adjustments when performing motor tasks. Improved agility skills can encourage individuals with Down syndrome to be more active and interactive in and with their environment, as well as decreasing their need to be dependent on a caregiver when performing activities of daily living.

Strength was assessed by Silva et al. (2017) using the 30-s sit-up and the standing broad jump. Results showed significant improvements for both outcomes within the experimental group compared with the control group at a 24-week intervention. However, no statistically significant results were found evaluating the handgrip strength test (Silva et al. 2017). Lin and Wuang (2012) also reported a significant improvement in strength in the following outcomes: standing long-jump, push-ups, sit-ups and v-ups within the experimental group after a 24-week intervention. Surprisingly, Wuang et al. (2011) found no significant change in strength between the experimental and control group after a 24-week intervention period using the BOT-2 strength subtest, whilst Lin and Wuang (2012) reported a significant change after a 6-week intervention period. Improved muscle strength will assist the individual with Down syndrome to perform more strenuous activities for longer time periods without getting tired. Individuals with Down syndrome often have hypermobile joints, ligament laxity as well as muscle hypotonia, which might decrease their stability when performing complicated functional movements (Hardee & Fetters 2017). Strengthening the muscle surrounding these joints will provide them with support and stability, as well as generating the force that will be available for them to complete the movement.

Assessment of coordination was carried out by both Silva et al. (2017), Álvareza et al. (2018) and Wuang et al. (2011). Silva et al. (2017) found only significant changes for the left-hand overhead throw. Besides a significant improvement in upper limb coordination, Wuang et al. (2011) found no significant results for the other coordination tests after 24 weeks of intervention. These studies had a similar frequency and duration of interventions. Álvareza et al. (2018) assessed coordination with the TGMD-2 manipulation subsection and found only significant improvement within-group changes in the experimental group. Berg et al. (2012) reported a significant improvement in upper limb coordination, as well as manual dexterity, after an 8-week intervention. All these study findings may lead to a decreased dependence on caregivers with activities of daily living, such as dressing, washing and eating. Successfully completing self-care activities without assistance may increase the feeling of self-worth and approval of peers. The Eurofit test battery used by Silva et al. (2017) (including the Flamingo balance test, shuttle run and the 30-s sit-up) has been shown to be a tool that is reliable when testing physical fitness in people with intellectual disabilities (Mac Donncha et al. 1999). Cabeza-Ruiz et al. (2019) found that the timed-up-and-go, hand grip test and the 30-s sit-up tests were reliable with good to high intra-class correlation coefficients in adults with Down syndrome (Cabeza-Ruiz et al. 2019).

### Limitations of included studies

One of the main limitations of the included studies was the inability of the researchers to blind the assessors and participants. The included studies had insufficient descriptions on the exact method of implementation of the interventions. All included studies had a wide variety of games that were available to the participants to choose from. However, none of the studies specified whether the participants were required to play all of the included games or whether they were given the option to choose one. Wuang et al. (2011) did not specify the games included for the experimental group. Rahman (2010) reported that there was no control over the intensity, amount of time and frequency of the home exercise techniques taught in the therapy sessions. Silva et al. (2017) and Ghafar and Abdelraouf (2017) reported that the small sample size of the study may have limited the chance to detect significant differences in some of the physical outcomes. Only Wuang et al. (2011) and Lin and Wuang (2012) had larger sample sizes (53 and 46 respectively). The rest of the included studies had small sample sizes, limiting the generalisability of the results.

### Limitations of our study

The inclusion criteria of this review led to two major limitations: Firstly, only seven of the studies complied with the inclusion criteria of this review, which may have an effect on the overall validity of the results because of the lack of available evidence. Secondly, articles that were not published in English were automatically excluded, potentially introducing a language bias. Furthermore, this review included only published studies, resulting in a publication bias.

Of the included studies, two were conducted over 24 weeks (Silva et al. 2017; Wuang et al. 2011), two were conducted over 8 weeks (Berg et al. 2012; Ghafar & Abdelraouf 2017), one over 6 weeks (Lin & Wuang 2012; Rahman 2010) and one over 5 weeks (Álvareza et al. 2018). This provided some difficulty with comparing the results of long-term follow-up, with four different intervention periods. Another limitation of this systematic review was that six of the studies (Álvareza et al. 2018; Berg et al. 2012; Ghafar & Abdelraouf 2017; Lin & Wuang 2012; Rahman 2010; Wuang et al. 2011) had a younger population (6–12 years) compared with Silva et al. (2017) who had an older population (18–60 years). As discussed earlier, balance weakens with aging and may be a reason why the balance outcome measure results differed greatly among these studies (Iwasaki & Yamasoba 2015). Finally, five of the included studies had small sample sizes within their studies, whilst Wuang et al. (2011) and Lin and Wuang (2012) had much larger sample sizes. This could possibly have an impact on the reliability of the results.

As a result of large difference in intervention periods, it was not possible to pool the data in a meta-analysis. Wuang et al. (2011) measured the outcomes after a 24-week intervention, and Lin and Wuang (2012) measured the outcomes after a 6-week intervention (three 35-min sessions per week for 6 weeks).

### Strengths of this review

A comprehensive, systematic search strategy was implemented, using nine computerised scientific databases. Also, each step of the review was completed independently by a reviewer and cross checked by another. The six included experimental studies were of high methodological quality ranging from 6/11 to 9/11 on the PEDro scale. The case study scored 6/8 on the JBI Critical Appraisal Checklist for case reports. Another strength of our review is the broad age range of the participants (6–60 years), making the data obtained in our review applicable to a broader population. However, this may have an impact on the validity of the results for our review, as the results are not applicable to a specific age group.

### Clinical implications

Clinicians are advised that it may be beneficial to use VRT, when available, in addition to standard physiotherapy or occupational therapy interventions for improving agility in individuals with Down syndrome, as it could be a valuable addition to standard physiotherapy or occupational therapy practice. Virtual reality therapy can also be used for balance training in a younger population, specifically children. The advised duration of intervention is 5–24 weeks. However, this relatively expensive electronic device may not be feasible in low- and middle-income countries or low-resource settings. The findings for both strength and coordination are inconclusive.

## Conclusion

The evidence of Level II, III-1 and IV does not favour the use of VRT, specifically Nintendo Wii, combined with physiotherapy or occupational therapy, over the use of standard physiotherapy or occupational therapy alone for motor proficiency. However, the results revealed that VRT was effective in improving agility within a five- or 24-week intervention. Furthermore, VRT may be effective in improving the strength within a 6 or 24-week period. Balance showed inconclusive results as a significant improvement was only seen in the child population and not in the adult population. Finally, results were inconclusive for coordination as not all studies showed significant improvements. However, upper limb and bilateral coordination improved significantly within a 24-week period. Further research should focus on frequent intervention sessions with regular follow-up assessments, as well as long-term follow-up, to investigate the carry-over effect of VRT. Although VRT is a valuable tool to include in a physiotherapy programme to increase agility in individuals with Down syndrome, as well as balance in children with Down syndrome, it cannot be used to replace standard physiotherapy. Clinicians are, therefore, advised to use VRT, when available, in addition to standard physiotherapeutic intervention.

## Appendix 1

**TABLE 1-A1:** Preferred Reporting Items for Systematic Reviews and Meta-Analyses checklist for systematic reviews and meta-analyses.

Section or topic	#	Checklist item	Page #
**Title**			
Title	1	Identify the report as a systematic review, meta-analysis, or both.	1
**Abstract**			
Structured summary	2	Provide a structured summary including, as applicable: background; objectives; data sources; study eligibility criteria, participants, and interventions; study appraisal and synthesis methods; results; limitations; conclusions and implications of key findings; systematic review registration number.	2–3
**Introduction**			
Rationale	3	Describe the rationale for the review in the context of what is already known.	6
Objectives	4	Provide an explicit statement of questions being addressed with reference to participants, interventions, comparisons, outcomes, and study design (PICOS).	7
**Methods**			
Protocol and registration	5	Indicate if a review protocol exists, if and where it can be accessed (e.g. Web address), and, if available, provide registration information, including registration number.	6
Eligibility criteria	6	Specify study characteristics (e.g. PICOS, length of follow-up) and report characteristics (e.g. years considered, language, publication status) used as criteria for eligibility, giving rationale.	8–9
Information sources	7	Describe all information sources (e.g. databases with dates of coverage, contact with study authors to identify additional studies) in the search and date last searched.	7–8
Search	8	Present full electronic search strategy for at least one database, including any limits used, such that it could be repeated.	App. B
Study selection	9	State the process for selecting studies (i.e. screening, eligibility, included in systematic review, and, if applicable, included in the meta-analysis).	9–10
Data collection process	10	Describe method of data extraction from reports (e.g. piloted forms, independently, in duplicate) and any processes for obtaining and confirming data from investigators.	10
Data items	11	List and define all variables for which data were sought (e.g. PICOS, funding sources) and any assumptions and simplifications made.	9
Risk of bias in individual studies	12	Describe methods used for assessing risk of bias of individual studies (including specification of whether this was done at the study or outcome level), and how this information is to be used in any data synthesis.	10
Summary measures	13	State the principal summary measures (e.g. risk ratio, difference in means).	12
Synthesis of results	14	Describe the methods of handling data and combining results of studies, if done, including measures of consistency (e.g. I^2^) for each meta-analysis.	N/A
Risk of bias across studies	15	Specify any assessment of risk of bias that may affect the cumulative evidence (e.g. publication bias, selective reporting within studies).	N/A
Additional analyses	16	Describe methods of additional analyses (e.g. sensitivity or subgroup analyses, meta-regression), if done, indicating which were pre-specified.	N/A
**Results**			
Study selection	17	Give numbers of studies screened, assessed for eligibility, and included in the review, with reasons for exclusions at each stage, ideally with a flow diagram.	10–11
Study characteristics	18	For each study, present characteristics for which data were extracted (e.g. study size, PICOS, follow-up period) and provide the citations.	14
Risk of bias within studies	19	Present data on risk of bias of each study and, if available, any outcome level assessment (see item 12).	N/A
Results of individual studies	20	For all outcomes considered (benefits or harms), present, for each study: (a) simple summary data for each intervention group (b) effect estimates and confidence intervals, ideally with a forest plot.	14–17
Synthesis of results	21	Present results of each meta-analysis done, including confidence intervals and measures of consistency.	N/A
Risk of bias across studies	22	Present results of any assessment of risk of bias across studies (see Item 15).	N/A
Additional analysis	23	Give results of additional analyses, if done (e.g. sensitivity or subgroup analyses, meta-regression [see Item 16]).	N/A
**Discussion**			
Summary of evidence	24	Summarise the main findings including the strength of evidence for each main outcome; consider their relevance to key groups (e.g. healthcare providers, users, and policymakers).	18–19
Limitations	25	Discuss limitations at study and outcome level (e.g. risk of bias), and at review-level (e.g. incomplete retrieval of identified research, reporting bias).	20–21
Conclusions	26	Provide a general interpretation of the results in the context of other evidence, and implications for future research.	18–19
**Funding**			
Funding	27	Describe sources of funding for the systematic review and other support (e.g. supply of data); role of funders for systematic review.	24

*Source:* Moher, D., Liberati, A., Tetzlaff, J. & Altman, D.G., 2009, ‘Preferred reporting items for systematic reviews and meta-analyses: The PRISMA statement’, *Annals of Internal Medicine* 151(4), 264–269

## Appendix 2

### Search terms used to identify the relevant articles.

#### Development of search strategy

The following nine computerised bibliographic databases were accessed and searched through the Stellenbosch Library services: MEDLine, Pubmed, Cochrane Library, PEDro, CINAHL, Science Direct, Scopus, Otseeker and Google Scholar. Stepwise documentation of the search process was done. Each database was independently searched by six researchers, thereby automatically cross-checking them. Each researcher independently identified relevant review titles from the databases and discussed the options with the other researchers. Preliminary searches within each database allowed for the elimination of unnecessary search terms, where the addition of key words did not yield varying results. Keywords included: Down syndrome, trisonomy-21, virtual reality, motor learning, Nintendo Wii, motor proficiency, motor performance, physiotherapy, physical therapy, exercises, physical fitness, functional mobility. The following search strategies for the various databases were developed according to the function of each database:

### Search strategies for various databases

#### Medline

**Example of search strategy**

1. ‘Down Syndrome’ AND ‘Motor Proficiency’
2. #1 AND ‘Nintendo Wii’
3. #1 AND ‘Virtual Reality’
4. ‘Down Syndrome’ AND ‘Motor Proficiency’ AND ‘Virtual Reality’
5. ‘Down Syndrome’ AND ‘Motor Proficiency’ AND ‘Physiotherapy’
6. ‘Down Syndrome’ AND ‘Motor Proficiency’ AND ‘Physical Therapy’
7. ‘Down Syndrome’ AND ‘Motor Proficiency’ AND ‘Virtual Reality’ AND ‘Physiotherapy’
8. ‘Down Syndrome’ AND ‘Motor Proficiency’ AND ‘Virtual Reality’ AND ‘Physical Therapy’

#### PUBMED

Limits applied to database

- Type of search: Advanced search
- Publication dates: Inception to April 2018, searches updated June 2018 and June 2020
- Publication types: Randomised or non-randomised Controlled/ Clinical Trials or pilot studies
- Population: Humans
- Language: English

**Example of search strategy**

1. ‘Down Syndrome’ AND ‘Motor proficiency’
2. #1 AND ‘Nintendo Wii’
3. #1 AND ‘Virtual Reality’
4. ‘Down Syndrome’ AND ‘Virtual Reality’
5. #1 AND ‘Virtual Reality’ AND ‘Physical therapy’

#### Cochrane Library

Limits applied to the database:

- Type of search: Simple and advanced search
- Publication dates: Inception to April 2018, searches updated June 2018 and June 2020
- Publication types: Randomised or non-randomised Controlled/ Clinical Trials or pilot studies

**Example of search strategy**

1. ‘Down Syndrome’ AND ‘Motor Proficiency’
2. #1 AND ‘Nintendo Wii’
3. #1 AND ‘Virtual Reality’
4. ‘Down Syndrome’ AND ‘Virtual Reality’ AND ‘Physical therapy’
5. ‘Down Syndrome’ AND ‘Nintendo Wii’ AND ‘Physical therapy’
6. #1 AND ‘Virtual Reality’ AND ‘Physical Therapy’

#### PEDro

Limits applied to the database:

- Type of search: Simple search
- Publication dates: Inception to April 2018, searches updated June 2018 and June 2020
- Publication types: Randomised or non-randomised Controlled/ Clinical Trials or pilot studies

**Example of search strategy**

1. ‘Down Syndrome’ AND ‘Motor Proficiency’
2. #1 AND ‘Virtual Reality’
3. #1 AND ‘Nintendo wii’
4. #1 AND ‘Virtual Reality’ AND ‘Physiotherapy’

#### Science Direct

Limits applied to the database:

- Type of search: Advanced search
- Publication dates: Inception to April 2018, searches updated June 2018 and June 2020

**Example of search strategy**

1. ‘Down Syndrome’ AND ‘Motor Proficiency’
2. #1 AND ‘Nintendo Wii’
3. #1 AND ‘Virtual Reality’
4. ‘Down Syndrome’ AND ‘Motor Proficiency’ AND ‘Nintendo Wii’
5. ‘Down Syndrome’ AND ‘Motor Proficiency’ AND ‘Virtual Reality’
6. ‘Down Syndrome’ AND ‘Motor Proficiency’ AND ‘Nintendo Wii’ AND ‘Physiotherapy’
7. ‘Down Syndrome’ AND ‘Motor Proficiency’ AND ‘Virtual reality’ AND ‘Physiotherapy’
8. ‘Down Syndrome’ AND ‘Motor Proficiency’ AND ‘Nintendo Wii’ AND ‘Physical Therapy’
9. ‘Down Syndrome’ AND ‘Motor Proficiency’ AND ‘Virtual reality’ AND ‘Physical Therapy’

#### Scopus

Limits applied to the database:

- Type of search: Advanced search
- Publication dates: Inception to April 2018, searches updated June 2018 and June 2020
- Publication type: Randomised or non-randomised Controlled/ Clinical Trials or pilot studies
- Language: English
- Subject Areas: Health Sciences

**Example of a search strategy**

1. ‘Down Syndrome’ AND ‘Motor proficiency’
2. #1 AND ‘Nintendo Wii’
3. #1 ’Virtual Reality’
4. #1 AND ‘Nintendo Wii’ AND ‘Virtual Reality’
5. #1 AND ‘Virtual Reality’ AND ‘Physiotherapy’

#### Google Scholar

**Example of a search strategy**

1. ‘Down Syndrome’ AND ‘Motor Proficiency’ AND ‘Nintendo Wii’
2. ‘Down Syndrome’ AND ‘Motor Proficiency’ AND ‘Virtual Reality’
3. #1 AND ‘Physiotherapy’
4. #2 AND ‘Physiotherapy’

#### Otseeker

**Example of a search strategy**

1. ‘Down Syndrome’
2. #1 AND ‘Motor Proficiency’
3. #1 AND ‘Virtual Reality’
4. #1 AND ‘Nintendo Wii’
5. #2 AND ‘Virtual Reality’
6. #5 AND ‘Physiotherapy’

#### CINAHL

**Examples of search strategy**

1. ‘Down syndrome’ AND ‘Motor Proficiency’
2. ‘Down syndrome’ AND ‘Virtual Reality’
3. ‘Down syndrome’ AND ‘Nintendo Wii’
4. #1 AND ‘Virtual Reality’
5. #1 AND ‘Nintendo wii’
6. #1 AND ‘Physiotherapy’

## Appendix 3

### Joanna Briggs Institute critical appraisal checklist for case reports

Author: Berg et al.      Year: 2012      Record Number: 01

**Table:**

	Yes	No	Unclear	Not applicable
Were patient’s demographic characteristics clearly described?	-	□	X	□
Was the patient’s history clearly described and presented as a timeline?	□	X	□	□
Was the current clinical condition of the patient on presentation clearly described?	X	□	□	□
Were diagnostic tests or assessment methods and the results clearly described?	X	□	□	-
Was the intervention(s) or treatment procedure(s) clearly described?	X	□	□	□
Was the post-intervention clinical condition clearly described?	X	□	□	□
Were adverse events (harms) or unanticipated events identified and described?	X	□	□	□
Does the case report provide takeaway lessons?	X	□	□	□

Overall appraisal: Include: **YES;** Exclude □ Seek further info □

Comments (Including reason for exclusion)

Good takeaway lesson and reinforces the overall message of our systematic review

## Appendix 4

**TABLE 1-A4:** Description of outcome measures.

Variable	Description
BOT-2	Bruininks-Oseretsky Test of Motor Proficiency (BOTMP) is similar to the Bruininks–Oseretsky Test of Motor Proficiency-Second Edition (BOT-2). The BOT-2 is a shorter version but still measures the same components as the BOTMP with the same activities. Rahman (2010) only made use of the balance subsection of the BOTMP, whilst Wuang et al. (2011) assessed all four of the motor proficiency components of the BOT-2. Fine manual control composite measures the motor skills involved in tasks requiring precise control of finger and hand movements. Manual coordination composite evaluates speed, dexterity, and coordination of upper extremities. Body coordination composite and the strength and agility composite are discussed below. The four composite scores are combined to yield a total motor composite score (Bruininks 2005). **Subsection – Balance** The patients were asked to kick a ball to determine the preferred leg. Thereafter the following tests had to be performed to assess balance: (1) standing on a line drawn on the floor on the preferred leg whilst looking at a target on the wall, (2) standing on a balance beam on the preferred leg whilst looking at target on wall, (3) standing as in item 2 but with closed eyes, (4) walking forward with normal stride on a line on the floor, (5) walking forward on a balance beam on the floor with a normal stride, (6) use heel-to-toe gait to walk forward on a line on the floor, (7) using heel-to-toe gait to walk forward on a balance beam, (8) patient walks forward on a balance beam with a normal stride and stepping over a response speed stick, held above the beam just under knee level, by the examiner (Bruininks 2005). **Subsection – Agility** BOT-2 has a Running speed and Agility subtest which contains five items: one-legged side hop, two-legged side hop, one-legged stationary hop, shuttle run and stepping sideways over a balance beam. The five test item scores were totalled to get an overall subtest score (Bruininks 2005). **Subsection – Strength** The Strength subsection of BOT-2 is designed to measure trunk, upper and lower body strength. It includes both 30 s push up, as well as sit-up test and a standing broad jump attempt (Bruininks 2005). **Subsection- Coordination** The BOT-2 assesses manual coordination composite that is classified into manual dexterity and upper-limb coordination subtests that evaluate reaching, grasping, and object manipulation, with the emphasis on speed, dexterity, and coordination of upper extremities. Body coordination composite is grouped into bilateral coordination and balance subtests that tap the balance and motor skills (Wuang et al. 2011). According to Wuang et al. (2011), the BOT-2 is a valid assessment tool for this population group if it has a satisfactory correspondence to other motor performance measures, namely the Peabody Developmental Motor Scale, Second Edition (Folio & Fewell 2000) and the Test of Visual Motor Skills-Revised (Gardner 1995).
Eurofit Test Battery	This is a physical fitness test consisting of numerous domains (Oja & Tuxworth 1995). It has been proven that this tool is exceptionally reliable when testing physical fitness in people with intellectual disabilities (Mac Donncha et al. 1999). Silva et al. (2017) used three of these domains as assessment tools to measure the motor proficiency components of their study group. **Flamingo balance test** This test is used to measure the total body balance. The patient had to remove their shoes and stand on a beam. The patient then performed a single leg stand on the preferred leg whilst flexing the other leg at the knee and holding the foot close to the buttocks. This began as soon as the patient let go of the instructor’s hand. The number of times that the patient lost balance within 60 s was recorded. The loss of balance is determined either when the patient falls off the beam or when they lose the position of the free leg. The time that the patient effectively maintained the single leg balance was also recorded. The test was terminated and a score of zero was given if the patient lost their balance more than 15 times in the first 30 s. **Shuttle run** The shuttle run test is used to assess running speed and agility. The test is performed by running back and forth between two beacons placed 5 m apart. The individual does this for a total of 50 m and on a smooth surface (Bechtol 1954). **30-second sit-up** 30-second sit-up test requires the patient to perform as many sit-ups as they can in a period of 30 s to objectively quantify abdominal strength (Bruininks 2005).
Handgrip test	The handgrip strength test is designed to replicate the grip strength required to hold objects such as the wii-remote. The test is completed using a dynamometer that measures grip strength in kilograms (Bechtol 1954).
Standing Broad Jump	Standing broad jump is used to measure explosive leg power. The test is executed with the participant standing behind a line drawn on the floor. The participant then jumps forward from a still standing position and the distance jumped is measured from the starting line to the back of the heels (Glencross 1966).
Beanbag Overhead Throw	The beanbag overhead throw involves throwing a beanbag over the ipsi-lateral shoulder in the direction of a hoop located at the centre of a gymnastic mat. It is performed standing 2 m away from the mat facing away from the mat. This tests spatial orientation and mental rotation (Carmeli et al. 2008).
Pediatric Balance Scale (PBS)	The Berg Balance Scale has been modified to create the PBS. The PBS is specifically used to assess balance in young children that present with mild to moderate disabilities, and it has already been established that the PBS is a reliable and valid tool for the measurement of balance (Franjoine et al. 2003). The test is standardised for children over the age of 4 years; thus it is an appropriate tool to use for the population of this study (Ghafar & Abdelraouf 2017). The following materials were used to asses balance: adjustable height bench, chair with back support and arm rest, stop watch, masking tape-1 inch wide, a step stool 6 inches in height, chalkboard eraser, yardstick and a small level.
Timed-up-and-go test	This test measures the time it takes for an individual to stand up from an armchair, walk 3 m, turn around, and sit back in the chair again. It was originally developed to measure the functional ability of elderly people who were at risk of falling. The timed-up-and-go test has been proven to be a reliable test when assessing the functional mobility of individuals with Down syndrome. This is a good test to use as it is easily re-producible (Nicolini-Panisson & Donadio 2014). Ghafar and Abdelraouf (2017) made use of this test and allowed each participant two trials. They used the average result of the two trials. This test has been proven to be reliable and valid with the measurement of balance in children with disabilities, as well as measuring balance in typically developing children (Podsiadlo & Richardson 1991).
Five-times-sit-to-stand test	The Five-times-sit-to-stand-test can be used to assess the ability to perform transitional movements and it is a reliable test to use for the measurement of balance (Posiadlo & Richardson 1991). In this test, the participants are asked to transition from sit-to-stand as quickly as possible for five repetitions with their arms folded across the chest. The height of the chair is set at 43 cm. A stopwatch was used to record the total time it took to complete the test (Ghafar & Abdelraouf 2017; Whitney et al. 2005). Five-times-sit-to-stand test was found to be a reliable and valid test for functional balance testing in children with mild to moderate cerebral palsy (Kumban et al. 2013).
TGMD-2	The TGMD-2 is a tool used to identify deficits in gross motor development in children between the age of 3 and 10 years, evaluating 12 skills, grouped into two categories, namely locomotor skills and object control skills (Álvareza et al. 2018). This test shows evidence of high reliability (Ulrich 2000).
Pressure Centre	Berg (2012) did a case study on an individual with Down syndrome. and they used the Biodex Biosway Balance System for the measurement of balance before and after intervention. The individual stands on the Biosway platform and is then asked to perform certain movements. Their postural sway is measured through the platform and feedback is given regarding the balance reaction on the individual. The individual was placed on the pressure centre with their eyes open and with their eyes closed to assess the balance. It has been found that this is a valid tool to use when assessing postural sway in progressively challenging double leg and single leg activities. However, it does not specify that this tool was specifically linked to children with any form of disability. https://www.hindawi.com/journals/bmri/2019/8185710/

TGMD-2, Test of Gross Motor Development, Second Edition.
